# Development of Plant-Based Multivalent Vaccine Candidates for SARS-CoV-2 and Influenza Virus Using Inactivated *Lactococcus*

**DOI:** 10.3390/vaccines13030254

**Published:** 2025-02-27

**Authors:** Dong-Sook Lee, Hasanul Banna, Heeyeon Kim, Md Rezaul Islam Khan, Hai-Ping Diao, Shi-Jian Song, Young-Eui Kim, Haeji Kang, Jungsang Ryou, Joo-Yeon Lee, Jang-Hoon Choi, Inhwan Hwang, Sehee Park

**Affiliations:** 1 Division of Acute Viral Disease, Center for Emerging Virus Research, National Institute of Infectious Diseases, National Institute of Health, Cheongju 28159, Republic of Korea; kmary2@korea.kr (D.-S.L.); hykim9269@korea.kr (H.K.); wlsdml7@korea.kr (Y.-E.K.); haeji.kang@korea.kr (H.K.); zenith@nih.go.kr (J.R.); nimbus@korea.kr (J.-H.C.); 2 School of Interdisciplinary Bioscience and Bioengineering, Pohang University of Science and Technology (POSTECH), Pohang 37673, Republic of Korea; banna@postech.ac.kr; 3 Department of Life Science, Pohang University of Science and Technology, Pohang 37673, Republic of Korea; mkhan46@mtu.edu (M.R.I.K.); diaohaiping@postech.ac.kr (H.-P.D.); songshijian@caas.cn (S.-J.S.); 4 Center for Emerging Virus Research, National Institute of Infectious Diseases, National Institute of Health, Cheongju 28159, Republic of Korea; ljyljy@nih.go.kr

**Keywords:** bacterium-like particle, influenza, SARS-CoV-2, neutralizing antibody, vaccine

## Abstract

Background/Objectives: Since December 2019, the COVID-19 pandemic, driven by SARS-CoV-2, has caused ~690 million infections globally, manifesting with mild to severe symptoms, including pneumonia. After reduced activity, seasonal influenza re-emerged in winter 2022, creating a “twindemic” with SARS-CoV-2. Co-infections have been associated with higher risks, such as increased ventilator use and mortality, emphasizing the need for dual-target vaccines. This study investigates plant-based vaccines produced using a bacterium-like particle (BLP) system from *Lactobacillus sakei* to co-target SARS-CoV-2 and influenza. Methods: DNA fragments of the SARS-CoV-2 Omicron BA.1 variant spike (S) protein and H1N1 virus hemagglutinin (HA) ectodomain were synthesized and used to create recombinant constructs introduced into *Agrobacterium*. Protein expression was analyzed using Western blot and Bradford protein assays. Six-week-old K18-hACE2 mice were immunized with these antigens and challenged with influenza, SARS-CoV-2, or both to assess viral load and lung pathology at various times. Results: The SARS-CoV-2 S protein and influenza HA protein were successfully expressed in *Nicotiana benthamiana* and demonstrated strong binding to BLPs. In mouse models (BALB/c and K18-hACE2), these vaccines elicited potent humoral and cellular immune responses, with high neutralizing antibody titers and increased IFN-γ levels. Vaccinated mice demonstrated protection against viral challenges, reduced lung viral loads, and improved survival. In cases of co-infection, vaccinated mice showed rapid recovery and effective viral clearance, highlighting the potential of vaccines to combat simultaneous SARS-CoV-2 and influenza infections. Conclusions: Our findings highlight the potential of BLP-based multivalent vaccines for dual protection against major public health threats.

## 1. Introduction

The COVID-19 pandemic, caused by severe acute respiratory syndrome coronavirus 2 (SARS-CoV-2), has hit worldwide. The emergence of this disease has adversely affected global health, resulting in the deaths of more than 7 million people [1]. In response to the COVID-19 pandemic, numerous companies have rapidly developed vaccines and therapeutic drugs to reduce infection rates and COVID-19-related mortality. Although these efforts have significantly mitigated the pandemic, COVID-19 cases and related deaths continue to be reported worldwide [1]. COVID-19 poses long-term health problems in humans. Since the World Health Organization (WHO) lifted the global emergency status of COVID-19 on 5 May 2023, SARS-CoV-2 cases have been increasing. This increase is attributed to the lifting of mask mandates and the easing of social activity restrictions. Despite vaccine and antiviral drug interventions, SARS-CoV-2 has spread worldwide with emerging novel variants emerging frequently. It remains unclear when the ongoing wave of COVID-19 will subside and when the severity of the situation will persist.

Several studies have shown that SARS-CoV-2 often co-occurs with other viruses, including hepatitis B and C viruses (HBV and HCV), influenza, and dengue viruses [2,3,4,5,6,7,8]. Notably, co-infection with SARS-CoV-2 and influenza virus was common during the early stages of the COVID-19 pandemic before the widespread adoption of measures such as mask-wearing and social distancing. With the recent easing of these safety measures, SARS-CoV-2 and seasonal influenza are likely to continue to co-circulate, potentially leading to annual epidemics. SARS-CoV-2 and influenza viruses are airborne viruses that preferentially infect alveolar type II cells and cause acute respiratory distress syndrome (ARDS). Notably, when an influenza virus co-infects with SARS-CoV-2, it can increase the expression of ACE2 in alveolar cells, thereby increasing the viral load of SARS-CoV-2. Furthermore, co-infection with SARS-CoV-2 and influenza viruses might provoke an inflammatory response, leading to severe damage, particularly in the respiratory tract [9]. Moreover, co-infection markedly diminished the levels of total IgG and neutralizing antibodies against both viruses [10]. This co-infection is also correlated with increased weight loss and a higher mortality rate, particularly during the flu season [11]. Consequently, there is an urgent need to develop a vaccine that can simultaneously protect against both SARS-CoV-2 and influenza virus.

Currently, several major pharmaceutical companies are developing combination vaccines against both COVID-19 and influenza [12,13]. These vaccines utilize either mRNA technology or recombinant proteins coupled with adjuvants [14,15] and are currently undergoing various phases of clinical trials. The primary components of these combination vaccines include key elements of both diseases, such as the hemagglutinin (HA) protein found in seasonal influenza strains and the spike (S) protein derived from SARS-CoV-2. However, seasonal influenza vaccines require annual updates due to antigenic variations. Therefore, there is an immediate need to create a combined vaccine for COVID-19 and influenza, offering an effective means of immunization against both illnesses.

Plants are well known for their role as sources of biologically active compounds. For example, compounds like quercetin, resveratrol, and curcumin have chemopreventive and therapeutic properties [16]. In addition, recently, plants have gained attention as cost-effective alternative platforms for antigen production [17]. Indeed, a wide range of antigens from different infectious viruses, including viral infections like HIV, Zika, and influenza have been produced [18,19,20]. They require low-cost facilities and have lower maintenance costs, making vaccine production more affordable. Plants can be grown on a large scale in open fields or controlled environments such as greenhouses, enabling rapid production expansion to meet rising demands, particularly during pandemics. Unlike mammalian or insect cell cultures, plants do not harbor human pathogens, thereby minimizing the risk of contamination with harmful viruses or prions. Additionally, plants have been shown to produce a diverse array of antigens, including complexes with multiple subunits, via the transformation of multiple genes. They can also undergo post-translational modifications similar to those in humans, which are crucial for the functionality of many antigens.

Bacterium-like particles (BLPs) are prepared from bacteria such as *Lactococcus lactis* or *Lactobacillus sakei* after treatment with 10% TCA and boiling to remove extracellular lipopolysaccharides, thereby exposing the peptidoglycan layers of the bacterial cell wall. As they lack genetic material, they are unable to induce illnesses. After fusion with a target antigen, antigens can be displayed on the surface of BLPs through a specific peptidoglycan-binding domain. It has been shown that the antigen–BLP complex is highly immunogenic compared to the antigen alone. Therefore, BLPs act as both adjuvants and carriers of antigens. Various types of cells have been used for BLP production, including gram-negative (e.g., *E. coli* and *Salmonella*) and gram-positive (e.g., *Lactococcus lactis* and *Lactobacillus sakei*) bacteria. Gram-positive bacteria are frequently employed to produce BLPs because of their generally recognized safety (GRAS) status. Overall, BLPs represent a promising avenue for vaccine development, offering numerous benefits over traditional approaches [20]. Their adaptability, safety, and capacity to induce strong immune responses make them appealing options for next-generation vaccines.

In our previous study, we introduced BLP-based HA vaccines for the avian influenza virus [21]. Mouse experiments showed that each BLP vaccine significantly increased the levels of specific neutralizing antibodies against their respective antigens. In this study, we selected *Nicotiana benthamiana* due to its high protein yield, ability to perform essential post-translational modifications like glycosylation, and rapid growth. Additionally, it is widely recognized for its compatibility with various expression systems over other species. We produced S and HA proteins in *N. benthamiana*. Protein extracts prepared from leaf tissues of *N. benthamiana* were mixed with BLP prepared from *Lactobacillus sakei* to induce the binding of plant-produced antigens to BLP. Subsequently, the BLP–antigen complex was purified by simple centrifugation. BLP vaccine combining S and HA was evaluated for its efficacy in vivo. These experiments demonstrated that immunization with the S-HA combination induced a robust immune response in mice, protecting against both influenza and SARS-CoV-2 infections. Our plant-based BLP vaccine, designed to be convenient and cost-effective, represents a promising new approach for mitigating respiratory diseases caused by SARS-CoV-2 and influenza.

## 2. Materials and Methods

### 2.1. Plasmid Construction

The DNA fragments of S encoding the ectodomain of the S protein (amino acid positions 16–1213) of the SARS-CoV-2 Omicron BA.1. variant and HA encoding the ectodomain (amino acid positions 17 to 530) of A/Victoria/2570/2019 H1N1 virus were chemically synthesized with restriction sites BamH1 and SpeI at 5′ and 3′ ends, respectively (Geneuniversal, New York, NY, USA). We chemically synthesized another DNA fragment consisting of Spe1:Linker:FD:Scpl7:His5:HDEL:XhoI (FD:Scpl7), in which the foldon domain (FD) encoded a trimerization motif of the foldon from bacteriophage T4 fibritin, and Scpl7 encoded a LysM domain for binding to the peptidoglycan of BLPs derived from *Lactobacillus sakei*, Scpl7 was derived from GH25_muramidase (aa positions 199–253, YP_009623604) of the Streptococcus phage CP-7. His5 and HDEL, ER retention motifs, were used as purification tags to retain recombinant proteins in the ER. The S and HA fragments digested with BamH1 and SpeI were ligated to the pTEX1L vector [20], which had been digested with the same restriction endonucleases and then fused to the Arabidopsis BiP leader sequence at the N-terminal region. Subsequently, the FD:Scpl7 fragment digested with SpeI and XhoI was ligated to BiP:S and BiP:HA in the pTEXL1 vector digested with the same restriction endonucleases to yield the full-length recombinant constructs, pBiP:S:FD:Scpl7 and pBiP:HA:FD:Scpl7. The pTEX1L vector harbors the MacT promoter and RD29B terminator [20].

### 2.2. Agroinfiltration for Transient Expression of Antigens in N. Benthamiana

The expression vectors pBiP:S:FD:Scpl7 and pBiP:HA:FD:Scpl7 were introduced into *Agrobacterium tumefaciens*. The *Agrobacterium tumefaciens* (GoldBio, St. Louis, MO, USA) was transformed via electroporation and plated onto LB agar (LPS Solution, Daejeon, Republic of Korea) containing two different kinds of antibiotics, kanamycin (50 μg/mL) (AG Scientific, San Diego, CA, USA) and rifampicin (50 μg/mL) (AG Scientific, San Diego, CA, USA). From the plate, a single colony was inoculated into 5 mL of LB medium containing the same two antibiotics and incubated overnight at 28 °C in a shaking incubator. The overnight culture was transferred to 50 mL of LB liquid medium with the same antibiotics and incubated for 16 h. Afterward, the cells were harvested by centrifugation (3500× *g*, 10 min, 25 °C) and resuspended in infiltration buffer. The infiltration buffer consisted of 10 mM MES (free acid, monohydrate, LPS-free solution, Daejeon, Republic of Korea) and 10 mM Mg(SO)_4_ (LPS Solution, Daejeon, Republic of Korea), pH 5.6. The cell suspension was adjusted to an OD600 of 0.8. Finally, acetosyringone (Sigma-Aldrich, St. Louis, MO, USA), a phenolic compound as an inducer of virus genes in Agrobacterium, was added to 200 μM and incubated at room temperature for 1 h before infiltration [22]. Infiltration was performed in two different ways; for a smaller scale, a 1 mL syringe without a needle was used, and for a large scale, vacuum infiltration was used. Plants grown for 5–6 weeks under normal growth conditions in a greenhouse were positioned in an inverted orientation within a vacuum chamber filled with *Agrobacterium* suspension, and leaf tissues were submerged in the *Agrobacterium* suspension. Vacuum pressures ranging from 50 to 400 mbar were applied for 30 or 60 s. After releasing the vacuum, the plants were recovered from the chamber and grown for an additional 5–7 days under normal growth conditions. The leaves infiltrated with Agrobacteria were collected at 3, 5, and 7 days post-infiltration (DPIf; to void confusion with the abbreviation “DPI” for days post-infection, this study uses “DPIf” to denote “days post-infiltration”) to assess the expression levels of the recombinant constructs. For animal immunization, recombinant proteins were produced from leaves harvested at 5 DPIf after vacuum infiltration.

### 2.3. Western Blot for Expression Analysis

Leaf tissues were homogenized with five volumes (*w*/*v*) of extraction buffer (50 mM Tris-HCl (LPS Solution, Daejeon, Republic of Korea), pH 7.5, 150 mM NaCl (LPS Solution, Rebublic of Korea), 0.1% [*v*/*v*] Tween 20 (LPS Solution, Daejeon, Republic of Korea), and 1x protease inhibitor cocktail (Sigma-Aldrich, St. Louis, MO, USA). The homogenate was centrifuged at 14,000× *g* for 10 min. The supernatant was saved as total soluble proteins. For the analysis of target protein expression, total proteins (10 μg) were separated by SDS-PAGE and followed by Western blot analysis using anti-His antibody (1:5000). Chemiluminescence (Amersham Pharmacia Biotech, Uppsala, Sweden) was used to visualize protein bands, and images were captured using an Amersham Imager 680 (AI680) analyzer (Amersham Pharmacia Biotech, Buckinghamshire, UK).

### 2.4. Antigen Binding and Purification Using cBLP

*Lactobacillus sakei* in its stationary phase was collected, resuspended in phosphate-buffered saline (PBS) (LPS Solution, Daejeon, Republic of Korea), and adjusted to an optical density of 1.0 at 600 nm. Half the volume of trichloroacetic acid (10%) (Sigma-Aldrich, St. Louis, MO, USA) was then added to the *Agrobacterium* suspension and boiled at 100 °C for 10 min. The boiled solution was centrifuged and the resulting pellet was washed three times with PBS to ensure the removal of TCA. The pellet was resuspended at 1.0 of OD_600_ in Tris buffer (50 mM Tris-HCl, pH 8, and 1 mM CaCl_2_) supplemented with trypsin (1 µg/mL final concentration, bovine pancreases) (Sigma-Aldrich, St. Louis, MO, USA). After incubation at 37 °C for 4 h, the bacterial suspension was boiled for 5 min to inactivate trypsin and subjected to centrifugation at 5000× *g* for 5 min. The pellet was washed three times with PBS and resuspended in PBS to prepare BLP. An aliquot (l mL, 1.0 at OD600, 10^8^ CFU) was mixed with varying amounts of total soluble proteins (TSPs) containing S or HA recombinant proteins in an incubation buffer consisting of an incubation buffer (50 mM Tris-HCl, pH 7.5, 150 mM NaCl, 0.1% Tween 20, 1 × protease inhibitor cocktail, and 10% glycerol) at room temperature for 10 min. After incubation, the samples were subjected to centrifugation at 10,000× *g* for 5 min. The supernatant and pellet fractions were separately collected. The pellet fraction containing BLP-bound proteins was washed three times with the incubation buffer for the immunization of the mice. The BLP–antigen complex pellet was washed three times with the incubation buffer for the immunization of the mice.

### 2.5. Cells and Viruses

The Madin–Darby canine kidney (MDCK) cells and Vero-E6 cells (CRL-1586) were obtained from the American Type Culture Collection (ATCC, Manassas, VA, USA). MDCK and Vero-E6 cells were cultured in Eagle’s Minimum Essential Medium (MEM, GenDEPOT, Katy, TX, USA) and in Dulbecco’s Modified Eagle’s Medium (DMEM) (GenDEPOT, TX, USA), respectively. The media were supplemented with 10% fetal bovine serum (FBS) (Gibco, Thermo Fisher Scientific, Waltham, MA, USA), 1% penicillin–streptomycin (P/S; 10,000 U/mL) (Gibco, Thermo Fisher Scientific, Waltham, MA, USA). hCoV19/Korea/KDCA447321/2021 (an Omicron variant) was acquired from the National Culture Collection for Pathogens at the Korea National Institute of Health (KNIH) and cultured in the Vero-E6 cells. A/Victoria/2570/2019 (H1N1 influenza vaccine strain) was purchased from the World Health Organization (WHO). This influenza virus was propagated in 10-day-old, embryonated specific pathogen-free (SPF) chicken eggs at 37 °C. After 48 h, the allantoic fluid containing the influenza virus was collected. Viral stocks were kept at −70 °C for future experiments. All experimental procedures using the SARS-CoV-2 and influenza viruses were performed in an animal biosafety level 3 (ABSL-3) facility at the KNIH.

### 2.6. Immunization of BALB/c

Six-week-old female BALB/c mice (OrientBio, Seongnam-si, Gyeonggi-do, Republic of Korea; *n* = 10/group, after 7 days of acclimation, five mice were randomly placed in each cage) were immunized three times with SARS-CoV-2 S and/or influenza HA antigens via the intramuscular (*i.m.*) route. The antigen doses were 1 µg and 5 µg of SARS-CoV-2 S or influenza HA protein and their mixtures (1 µg + 1 µg and 5 µg + 5 µg, respectively) without adjuvant. After being primed with each vaccine antigen, the mice were boosted twice at 14-day intervals. On 14, 28, and 42 dpi, blood samples were collected from each mouse for measuring neutralizing antibody titers against SARS-CoV-2 spike and influenza HA proteins, respectively.

### 2.7. Human ACE Transgenic Mice Immunization and Challenge

Six-week-old female K18-hACE2 (The Jackson Laboratory, Bar Harbor, ME, USA) (after 7 days of acclimation, five mice were randomly placed in each cage) mice were housed in isolators in a biosafety level 3 (BL3) laboratory at the Korea National Institute of Health (KNIH) during the experiments. For the virus challenge experiments, mice (*n* = 23) were immunized via the *i.m.* route with SARS-CoV-2 and influenza antigens, administered at concentrations of 1, 2.5, and 5 μg in equal proportions. Mice received a booster immunization two weeks following the initial dose. On day 28 post-primary immunization, the mice were anesthetized and subsequently challenged intranasally with influenza virus, SARS-CoV-2 (Omicron BA.1 variant), or a combination of both pathogens. Lung tissues were collected from three mice at 3, 5, and 7 days post-infection (DPI) to measure viral titers and analyze histopathological changes through plaque assays. The remaining mice were monitored for a 14-day period to track body weight fluctuations and survival rates.

### 2.8. Enzyme-Linked Immunosorbent Assay (ELISA)

Total IgG antibodies against the SARS-CoV-2 and influenza viruses were measured using the antisera collected from each immunized mouse (13, 27, and 41 days post-immunized antisera). Briefly, purified recombinant S (0.5 µg/well) or HA (0.5 mg/well) protein in a carbonate buffer was coated in 96-well plates at 4 °C. The next day, the plates were washed three times with PBS containing 0.05% Tween 20 (PBS-T), and 5% skim milk-PBS was used to block the plates. The serially two-fold diluted mouse antisera in the blocking buffer (1:50 dilution) were added to the plates for overnight incubation at 4 °C. Then, the plates were washed three times again, and HRP-conjugated anti-mouse IgG (Cell Signaling Technology, Danvers, MA, USA) in the blocking buffer was applied for 1 h. Color changes in the plates were observed for 15 min with o-phenylenediamine dihydrochloride (OPD) (Sigma-Aldrich, St. Louis, MO, USA) followed by sulfuric acid treatment using an ELISA plate reader (BioTek, Winnoski, VT, USA) at 490 nm absorbance. Antibody titers were recorded as the reciprocal of the highest dilution that resulted in a signal at least twice the background level.

### 2.9. Enzyme-Linked Immunosorbent Spot

The expression of interferon-gamma (IFN-γ), interleukin-4 (IL-4), and granzyme B in immunized mice was evaluated using an ELISPOT assay, following the manufacturer’s protocol (R&D Systems, Minneapolis, MN, USA). Splenocytes were harvested from both immunized and control mice and adjusted to a concentration of 5 × 10^5^ cells per well. These cells were stimulated at 37 °C for 48 h with recombinant 2019-nCoV spike S protein or HA protein from A/Victoria/2570/2019 (Sino Biological, Beijing, China). After incubation, the plates were washed and incubated with biotinylated polyclonal antibodies specific to IFN-γ, IL-4, or granzyme B for 2 h at 4 °C. Following a PBS-T wash, streptavidin conjugated to HRP was applied and incubated at room temperature for 1 h. Cytokine secretion spots were measured after a 36 h co-culture at 37 °C under 5% CO_2_, according to the manufacturer’s guidelines. Spot images were captured, and the number of spots per well was quantified using an automated ELISPOT reader (Bioreader 4000; BIOSYS GmbH, Karben, Germany). Results were expressed as spot-forming cells (SFC) per 5 × 10^5^ cells.

### 2.10. Plaque Reduction Neutralization Assay

Neutralizing antibody titers against SARS-CoV-2 were measured using a plaque reduction neutralization test (PRNT) in a 12-well plate. First, the mouse antisera were inactivated at 56 °C for 30 min and serially two-fold diluted in 2% FBS-1% P/S-DMEM (FBS, P/S: Gibco, Thermo Fisher Scientific, Waltham, MA, USA; and DMEM: GenDEPOT, Baker, TX, USA). Equal volumes of the diluted sera and virus suspension (~50 PFU/100 µL) were mixed and maintained at 37 °C in a 5% CO_2_ incubator for 1 h. The mixture was then applied to Vero-E6 cells at 37 °C for 1 h. After incubation, the mixture was removed, and the cells were overlaid with DMEM supplemented with 2% FBS and 1% P/S or MEM supplemented with 2% BSA (Yourlab, GeorgiaChem, Seongnam-si, Gyeonggi-do, Republic of Korea) and 1% P/S, along with 1% Sea-Plaque agarose (Lonza, Basel, Switzerland). After 72 h incubation at 37 °C, the cells were fixed with 4% paraformaldehyde (Biosesang, Seongnam-si, Gyeonggi-do, Republic of Korea) and stained with 1% crystal violet (Sigma-Aldrich, MO, USA). The 50% neutralization titers (NT50) were determined based on the number of visible viral plaques using the Reed and Muench method [23].

### 2.11. Microneutralization Assay

A microneutralization (MN) assay was performed following a standard protocol, as described previously [24]. Briefly, heat-inactivated sera were treated with a receptor-destroying enzyme (RDE) (Denka Seiken, Tokyo, Japan) and then reacted with turkey red blood cells. The RDE-treated sera were serially diluted and incubated with 100 TCID_50_/mL of the influenza virus (A/Victoria/2570/2019) for 1 h. MDCK cell monolayers (2 × 10^4^ cells/well), rinsed with PBS, were exposed to the serum–virus mixtures for 1 h. After removing the inoculum, the MDCK cells were incubated overnight in media containing TPCK–trypsin (for the influenza virus, 2 mg/mL) (Gibco, Thermo Fisher Scientific, Waltham, MA, USA). The cells were subsequently rinsed with PBS, fixed in 80% acetone at room temperature for 10 min, and allowed to air dry. Plates were washed with PBS-T, followed by the addition of mouse monoclonal antibodies targeting the influenza nucleoprotein (NP), which were diluted in the blocking buffer. After incubation for 1 h, the plates were washed again with PBS-T, and an HRP-conjugated anti-mouse IgG antibody (Cell Signaling, Technology, Danvers, MA, USA) was incubated for another 1 h. After an additional PBS-T washing step, an OPD substrate was applied, and the reaction was terminated by adding 0.5 M sulfuric acid. Absorbance was measured at 490 nm using a microplate reader (BioTek, VT, USA).

### 2.12. Virus Titer in the Mouse Lungs

The lungs of the challenged mice were homogenized in 1 mL of medium, and the supernatants were obtained after centrifugation at 6000× *g* for 20 min at 4 °C. The supernatants were then used for viral titration. Viral titers of the SARS-CoV-2 and influenza viruses were determined using the plaque assay. Briefly, Vero E6 (for SARS-CoV-2) and MDCK (for the influenza virus) cells were prepared at a density of 2 × 10^5^ cells/well in 12-well plates. The confluent cells were washed with PBS and inoculated with serial 10-fold dilutions of the lung supernatants. After incubation at 37 °C for 1 h, the inoculum was discarded from the cells, and the cells were overlaid with 1% DMEM agarose containing 1% P/S (for SARS-CoV-2) and 0.6% MEM agarose supplemented with 1 mg/mL TPCK-treated trypsin (for the influenza virus, 2 mg/mL) (Gibco, Thermo Fisher Scientific, Waltham, MA, USA), respectively. The plates were then maintained at 37 °C for 72 h and fixed with 4% formaldehyde for at least 2 h. The cells were stained with 1% crystal violet, and visible viral plaques were counted.

### 2.13. Histopathological Examination

Whole lung tissues were collected from both mock and virus-infected mice at 4 DPI for histopathological examination. Briefly, the mice lungs were treated with 1% neutral-buffered formalin. Then, the lungs were embedded in paraffin for tissue section. The sectioned lung tissues were stained with hematoxylin and eosin (H&E) and examined under microscopy.

### 2.14. Statistical Analysis

All data were analyzed using GraphPad Prism software (version 8.0). Student’s *t*-test, and the two-way ANOVA method, and analysis of variance was used to calculate the *p*-values. “*” indicates *p* < 0.05, “**” indicates *p* < 0.01, and “***” indicates *p* < 0.001.

## 3. Results

### 3.1. Design of S and HA Recombinant Proteins and Their Expression in N. benthamiana

To produce an effective bivalent vaccine against both SARS-CoV-2 and influenza, we expressed recombinant antigens in plants and used BLP as a vehicle to deliver the recombinant antigens, the S protein of SARS-CoV-2, and the HA of influenza during vaccination. Antigens displayed on the surface of BLPs induce a strong immune response with or without any adjuvant [21]. To express antigens in plants and explore the BLP platform for vaccine production, we designed recombinant genes for the S protein of SARS-CoV-2 and the HA of influenza H1N1. To express antigens in plants, we used the Arabidopsis BiP leader sequence for ER targeting, followed by the entire ectodomain of the S protein or HA. Subsequently, the foldon motif from bacteriophage T4 fibritin [25] was included to induce trimer formation in both proteins because they are trimers in their native form. To achieve high antigen binding to BLP, we fused the LysM domain of Scpl7 to a cell wall-binding motif [22,26]. Additionally, a purification tag (His5) and an ER retention motif (HDEL) were added to the C-terminus of Scpl7. Thus, the recombinant S and HA genes had the domain structures BiP:S:FD:Scpl7:His:HDEL (S) and BiP:HA:FD:Scpl7:His:HDEL (HA), respectively. For expression, we used the pTEX1L vector with the MacT promoter and RD29B terminator [21]. The pTEX1L binary vector, derived from pCAMBI1300, contained *P38* (turnip crinkle virus coat protein gene) as a gene silencing repressor for high-level expression of the target gene.

**Figure 1:**
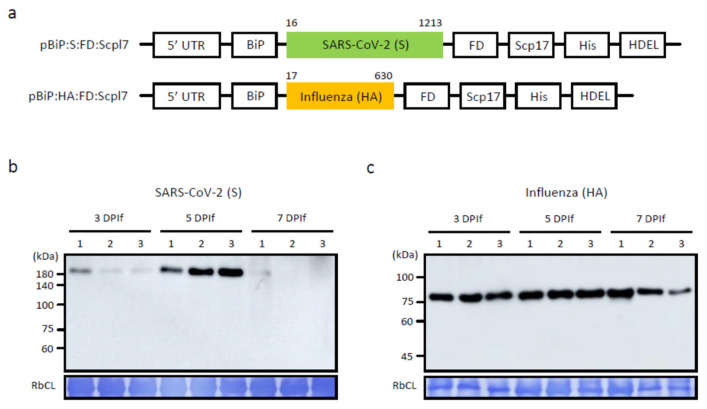
Expression of SARS-CoV-2 S and influenza HA proteins in *N. benthamiana*. (**a**) Schematic presentation of constructs for SARS-CoV-2 S and influenza HA proteins. The ectodomains of both S (16 to 1213) and HA (17 to 530) proteins were used for the expression of recombinant proteins in *N. benthamiana*. 5′ UTR, the untranslated region of Arabidopsis AT1G58420; BiP, the leader sequence of Arabidopsis BiP; FD, foldon; Scpl7, a LysM domain; His, 5 His residues as a purification tag; HDEL, an ER retention motif. (**b**,**c**) Expression of S and HA proteins in *N. benthamiana*. The recombinant constructs for S (**b**) and HA (**c**) were introduced into *Agrobacterium tumefaciens* GV3101. Agrobacterial suspensions (0.8 at OD_600_) were injected into leaf tissues of *N. benthamiana* using a syringe without a needle. Plants were further grown for an additional 3, 5, or 7 days. Total leaf extracts were prepared from harvested leaf tissues and analyzed by Western blotting using anti-His antibody. RbcL, the loading control was stained with Coomassie brilliant blue.

We examined the expression of *S* and *HA* constructs in *N. benthamiana* following *Agrobacterium tumefaciens*-mediated infiltration. Leaves were harvested on various days post-infiltration (DPIf), and the total protein extracts were analyzed by Western blotting using an anti-His antibody. The recombinant S protein displayed distinct bands at approximately 180 kDa, consistent with a previous report [27]. The HA protein exhibited a band at 80 kDa, which was larger than the calculated molecular weight, but similar to previous findings [28,29]. The expression levels of both constructs peaked at 5 DPIf compared to 3 and 7 DPIf (Figure 1).

### 3.2. Binding of S and HA Proteins to BLP and Preparation for Animal Immunization

**Figure 2:**
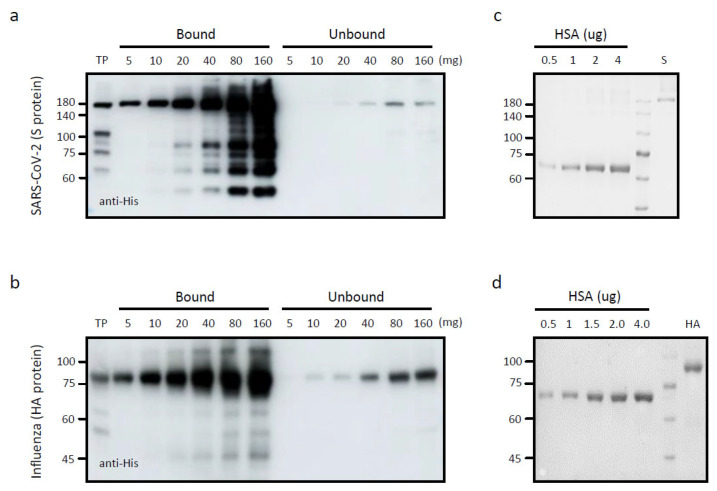
Binding of S and HA proteins to BLP. (**a**,**b**) Binding of S (**a**) and HA (**b**) proteins to BLP. Total protein extracts prepared from leaf tissues were mixed with l mL of BLP prepared from 10^8^ CFU, followed by incubation at room temperature for 30 min. After centrifugation, the supernatant (Unbound) and pellet (Bound) fractions were separated and analyzed by Western blot and CBB staining. (**c**,**d**) Estimation of S and HA proteins used for immunization. According to the results of (**a**,**b**), we prepared an antigen/BLP complex for immunization at the condition of 4 and 5 mg of S and HA proteins, respectively, at 10^8^ CFU. The amount of S and HA proteins bound to BLP was estimated by SDS-PAGE followed by CBB staining. The known amounts of HSA were loaded as a reference for the protein amount.

We assessed the binding affinities of both S and HA for BLP. Varying amounts of total protein extracts from the infiltrated leaves were incubated with equal amounts of BLP. Following incubation, the BLP-bound proteins were pelleted by centrifugation. The pellet fraction was washed thrice with PBS to remove unbound proteins. BLP-bound proteins were analyzed by Western blotting after SDS-PAGE (Figure S1). Both proteins exhibited a strong binding affinity for BLP. The amount of S and HA proteins bound to BLP derived from 10^8^ cells was approximately 4 µg and 5 µg, respectively (Figure 2).

For the immunization of animals, we prepared a BLP–antigen complex using 20 g of infiltrated leaves harvested at 5 DPIf. The total soluble protein extracts were mixed with appropriate quantities of BLP and incubated at room temperature for 10 min. After incubation, the samples were centrifuged at 10,000× *g* for 5 min to separate the pellet containing BLP-bound proteins from the unbound fraction. The pellet containing the BLP–antigen complex was washed three times with the incubation buffer. The final pellet containing the BLP–antigen complex was resuspended in 2 mL of PBS. To quantify the amount of antigen bound to BLP, the BLP–antigen complex along with varying amounts of human serum albumin (HAS) was separated by SDS-PAGE (10 µL load from 2 mL) and stained with Coomassie brilliant blue. Antigen quantity was determined by comparison with HSA loaded onto the same gel. The concentrations of S and HA proteins bound to BLP were 0.06 µg/µL and 0.25 µg/µL, respectively. The concentration was further adjusted by modifying the final volume of the BLP–antigen complex before injection into the mice.

### 3.3. Assessment of Immune Responses of Multivalent BLP Vaccination in BALB/c Mice

**Figure 3:**
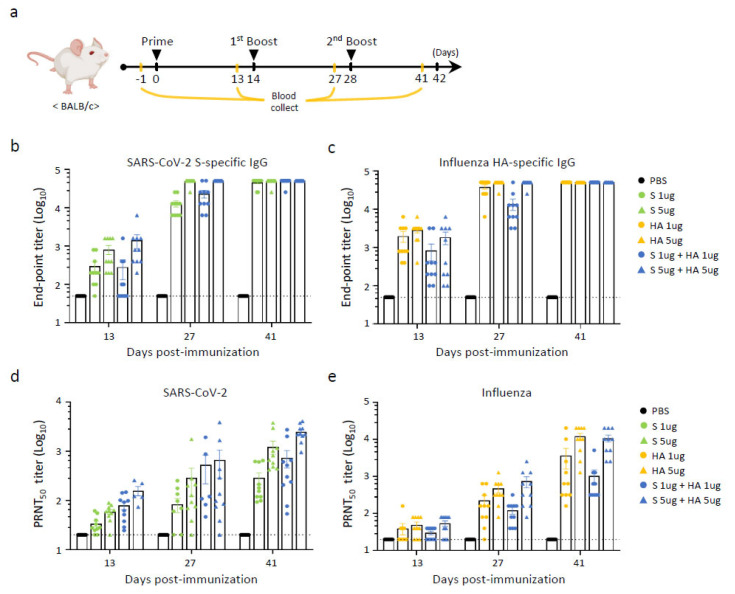
Humoral immune response of divalent BLP vaccination in BALB/c mice. (**a**) Experimental design showing vaccination schedule and sample collection. The BLP vaccine candidates include iLact-tSP and iLact-tHA. iLact-tSP represents the S protein of SARS-CoV-2, while iLact-tHA represents the HA protein of influenza. Mice were injected intramuscularly with a combination of either 1 or 5 μg of iLact-tSP and iLact-tHA three times in two-week intervals. iLact-tSP or iLact-tHA were also injected at dose 1 or 5 µg to compare the effects of a single BLP. PBS was used as a negative control. Mice serum samples were collected two weeks following each immunization. (**b**) The endpoint titers of IgG antibodies were measured using ELISA plates coated with recombinant SARS-CoV-2 spike protein. (**c**) Similarly, the endpoint titers of IgG antibodies against recombinant influenza HA antigen were assessed using ELISA plates. (**d**) Neutralizing antibody titers specific to the SARS-CoV-2 spike protein were determined via a 50% plaque reduction neutralization test (PRNT_50_) at 14, 28, and 42 days post-immunization. (**e**) Neutralizing antibody titers against influenza virus were evaluated using a microneutralization (MN) assay. All experiments were performed independently in duplicate, with 10 mice per group for each experiment (*n* = 10/group).

To evaluate whether the BLP multivalent vaccine could induce an immune response, SARS-CoV-2 S and influenza HA proteins were administered to mice (*n* = 10/group). To determine the optimal antigen concentration, S and HA proteins were combined in quantities of 1 or 5 μg each. Additionally, single antigens at 1 or 5 μg were inoculated into mice for comparison. The BLP antigens were delivered intramuscularly without adjuvants. The immunized group was compared to a control group injected with PBS only. Six-week-old mice were boosted twice at two-week intervals, following the initial immunization, and sera were collected one day before each immunization (Figure 3a).

ELISA plates coated with S or HA proteins were used to assess the presence of SARS-CoV-2 S-specific IgG or influenza HA-specific IgG antibodies. Two weeks after the initial vaccination (prime), antigen-binding antibodies targeting S and HA were detected in all groups of mice, including those administered a single antigen. At two weeks after the first boosting, the antibody responses increased in a dose-dependent manner. Following the second booster, antibody titers appeared to reach a saturated endpoint in all the immunized groups (Figure 3b,c).

To determine the neutralizing antibody titers against SARS-CoV-2 Omicron BA.1 in the BLP-immunized mice, we performed the plaque reduction neutralization test (PRNT). The same sera used for the ELISA were used in this test. Two weeks after the initial booster immunization, all vaccinated groups exhibited the induction of neutralizing antibodies (Figure 3c). Notably, the group that received a combination of SARS-CoV-2 S and influenza HA proteins displayed elevated levels of nAbs compared to the group immunized solely with SARS-CoV-2 S protein (Figure 3d). Furthermore, the group immunized with 5 μg of antigen exhibited a greater nAb response than that immunized with 1 μg of antigen. Simultaneously, nAbs against influenza HA protein were measured using ELISA with the same serum (Figure 3e). As the number of immunizations increased, the titer of nAbs against the influenza HA protein also increased. Sera from mice administered with 5 μg of HA antigen showed higher titers than those from mice administered with 1 μg of HA antigen.

### 3.4. Humoral and Cellular Immune Responses of Multivalent BLP Vaccination in K18-hACE2 Mice

**Figure 4:**
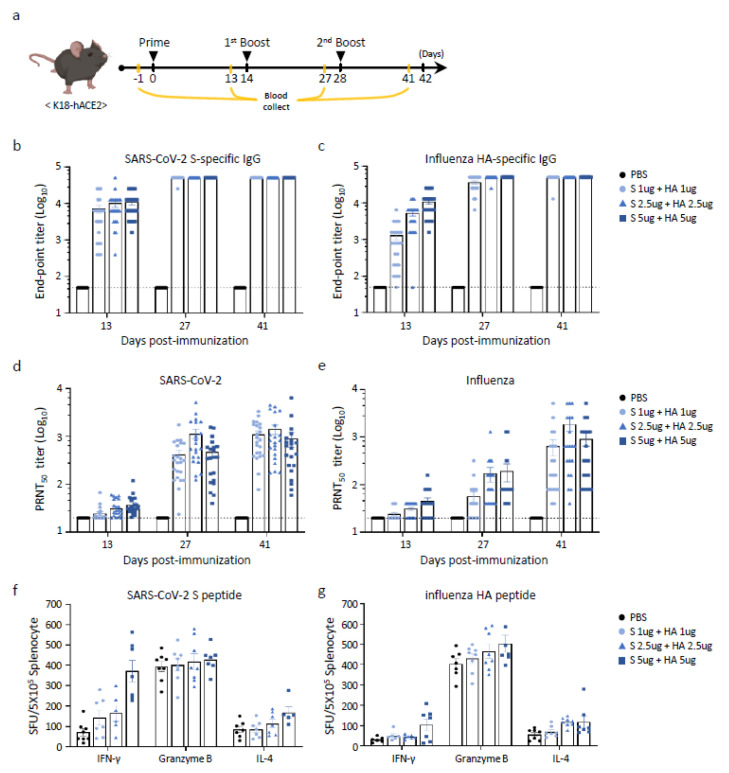
Humoral immune response of divalent BLP vaccination in K18 hACE2 mice. (**a**) Experimental design showing vaccination schedule and sample collection. Mice were intramuscularly administered a mixture of iLact-tSP and iLact-tHA at doses of 1, 2.5, and 5 μg three times at two-week intervals. Mouse serum samples were collected two weeks after each immunization. (**b**) Endpoint IgG antibody titers were evaluated using ELISA plates coated with recombinant SARS-CoV-2 S antigen. (**c**) Similarly, endpoint IgG antibody titers were assessed with ELISA plates coated with recombinant influenza HA antigen. (**d**) Neutralizing antibody titers against SARS-CoV-2 were measured using a 50% plaque reduction neutralization test (PRNT_50_) on days 13, 27, and 41 post-immunization. (**e**) Neutralizing antibody titers specific to the influenza virus were determined via microneutralization (MN) assay. (**f**) Cytotoxic T-cell responses in splenocytes were analyzed 14 days after the second booster dose. The production of IFN-γ and granzyme B was quantified using an ELISPOT assay after stimulation with recombinant SARS-CoV-2 S protein. (**g**) IFN-γ and granzyme B secretion levels were also measured in an ELISPOT assay following stimulation with recombinant influenza HA protein. The data are presented as the mean ± SEM. Each experiment was performed independently in duplicate with 10 mice per group (*n* = 10).

We also investigated whether immune responses against SARS-CoV-2 and influenza were induced in transgenic K18-hACE2 mice expressing human ACE2. The concentrations of SARS-CoV-2 and influenza were subdivided into 1, 2.5, and 5 μg per sample, mixed equally, and subsequently administered via the *i.m.* route into these transgenic mice. Sera were collected 2 weeks after prime and boost immunizations, and nAb production was measured (Figure 4a). After priming, SARS-CoV-2 S-specific antibodies increased in an antigen dose-dependent manner, reaching saturation after the first booster immunization (Figure 4b). HA-specific antibodies were also induced to saturation levels following the first boost, similar to SARS-CoV-2 S-specific antibodies (Figure 4c). Endpoint titers in K18-hACE2 mice were approximately 10-fold higher than those in BALB/c mice. In contrast, SARS-CoV-2 S-specific antibodies in K18-hACE2 mice were barely induced after priming; nAbs were confirmed only after the first booster immunization, with an average titer of 76 (range, 35–156). The nAb levels after the first boost in K18-hACE2 mice were comparable to those in BALB/c mice (Figure 4d). We also assessed the effects of nAbs on influenza HA. The levels of nAbs against HA after priming and the first boost were comparable to those in BALB/c mice. However, the nAb levels after the secondary boost were lower than those in BALB/c mice (Figure 4e).

The cellular immune responses elicited by BLP (SARS-CoV-2 + influenza) were evaluated using an ELISPOT assay. Fourteen days after the second boost, the mice (*n* = 8 per group) were sacrificed, and splenocytes were collected. Splenocytes were cultured with recombinant S or HA antigens as stimulators. Subsequently, the induction of intracellular interferon γ (IFN-γ) and interleukin-4 (IL-4) was measured (Figure 4f,g). Compared to the PBS-treated group, all BLP-immunized groups exhibited higher secretion levels of both IFN-γ and IL-4, suggesting a stronger cytotoxic T-cell response compared to the PBS-treated group. These findings indicate that BLP could be utilized not only as a vaccine candidate targeting a single antigen (SARS-CoV-2 S or influenza HA protein) but also as a multivalent vaccine candidate containing both SARS-CoV-2 S and influenza HA proteins.

### 3.5. Evaluation of Protective Efficacy of Multivalent BLP Against SARS-CoV-2 and Influenza Viruses

**Figure 5:**
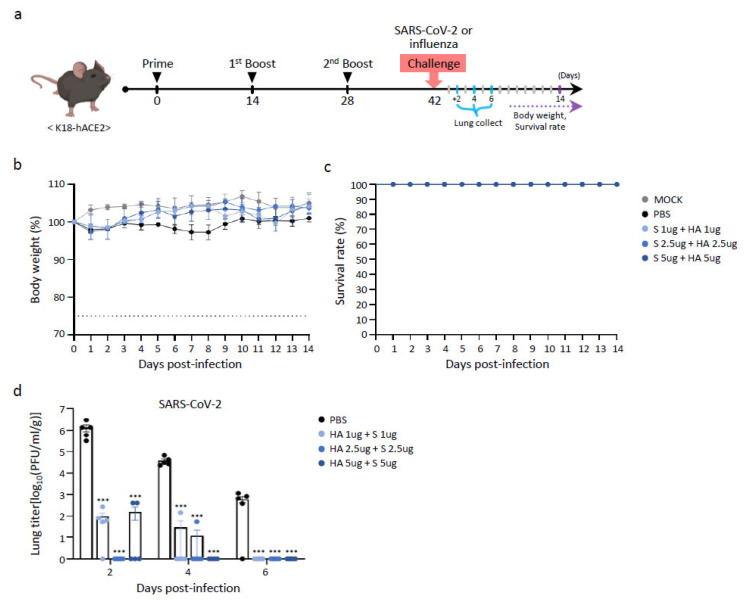

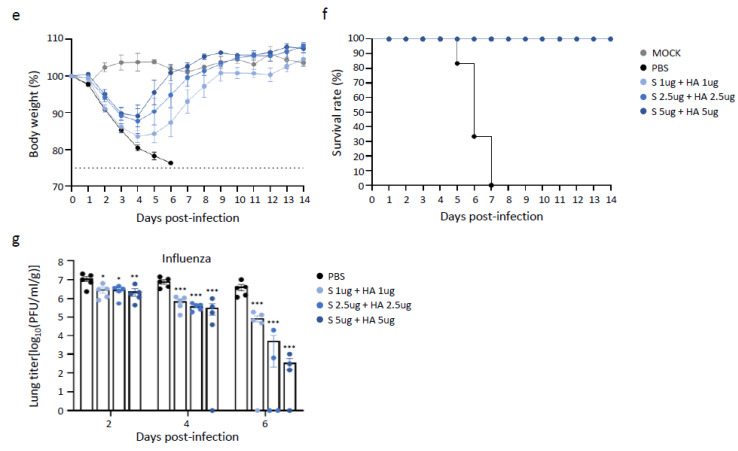
Evaluation of the protective effects of a multivalent BLP vaccine candidate against challenges with SARS-CoV-2 and influenza in K18-hACE2 mice. (**a**) Schematic representation of the experimental design, including vaccine immunization and subsequent viral challenges (SARS-CoV-2 or influenza). Mice were intramuscularly administered a mixture of iLact-tSP and iLact-tHA at doses of 1, 2.5, and 5 μg three times at two-week intervals. Two weeks after the 2nd boost immunization, mice were challenged with SARS-CoV-2 (2 × 10^5^ PFU) or influenza (2 × 10^5^ PFU). After influenza virus challenge, (**b**) body weight changes and (**c**) survival were monitored for 2 weeks (*n* = 7). A weight loss exceeding 25% of the initial body weight is deemed an experimental endpoint. (**d**) Mice (*n* = 5) were euthanized on 2, 4, and 6 DPI and the viral titers in the lungs were determined in a conventional plaque assay on MDCK cells. After SARS-CoV-2 challenge, (**e**) body weight changes and (**f**) survival were monitored for 2 weeks (*n* = 7). Mice (*n* = 5) were euthanized on 2, 4, and 6 DPI and the (**g**) viral titers in the lungs were determined in a conventional plaque assay on Vero E6 cells. Data are expressed as the mean ± SEM. Experiments were conducted independently. The statistical significance of differences between groups was calculated by two-way ANOVA with Tukey’s multiple comparisons test (* *p* < 0.05, ** *p* < 0.01, *** *p* < 0.001). Dotted lines reflect the assay limit of quantitation.

We investigated whether BLP-immunized K18-ACE2 mice exhibited protective efficacy against SARS-CoV-2 and influenza challenge. BLP combination sets for SARS-CoV-2 and influenza were prepared by mixing SARS-CoV-2 S with influenza HA BLP at doses of 1, 2.5, and 5 μg. K18-hACE2 mice (*n* = 23) were inoculated three times with either PBS or one of the three BLP combination sets at 2-week intervals via the *i.m.* route (Figure 5a). Fourteen days after the second booster immunization, K18-hACE2 mice were challenged with either PBS or 2 × 10^5^ PFU of influenza virus (A/Victoria/2570/2019) via the intranasal (*i.n.*) route. Body weight change (%) and survival were monitored over a 14-day period (Figure 5b,c). The PBS-treated mice gradually lost weight after the influenza virus challenge, with all mice succumbing at 6 DPI. Mice immunized thrice with the BLP combination sets exhibited weight loss until 4 DPI, after which they began to recover, regaining weight to levels similar to those in the PBS-treated group. Viral titers in the lungs after the challenge were also measured. Lung tissues were collected from three mice euthanized at 2, 4, and 6 DPI. Viral loads in the lungs of the BLP-immunized group were consistently lower than those in the PBS-treated group at all time points (Figure 5d).

To evaluate the protective effects against SARS-CoV-2, K18-hACE2 mice that received three immunizations with BLP were exposed to SARS-CoV-2 Omicron BA.1 (NC928) at a dose of 2 × 10^5^ PFU. Changes in body weight (%) and survival rates were tracked over a 14-day period (Figure 5e,f). In agreement with earlier reports [17,18], mice in all vaccinated groups showed no significant weight loss compared to the PBS-treated control group. Simultaneously, viral titers were measured in lung tissue obtained from mice infected with SARS-CoV-2 (Figure 5g). Lung tissues were collected from three mice euthanized at 2, 4, and 6 DPI. In the lungs of PBS-treated mice, viral titers increased to 10^6^ PFU/mL at 2 DPI and then gradually decreased to 10^3^ PFU/mL at 6 DPI. In contrast, the viral titers in the lungs of mice immunized three times with BLP were reduced to 10^2^ PFU/mL, even with a low vaccine dose (1 μg of S + 1 μg of HA). Remarkably, no viruses were detected at 6 DPI. These results demonstrated the efficacy of the BLP vaccine against SARS-CoV-2. Collectively, these data suggest that the BLP vaccine protected against SARS-CoV-2 and influenza.

### 3.6. Protective Efficacy of Multivalent BLP Against Co-Infection with SARS-CoV-2 and Influenza Viruses

**Figure 6:**
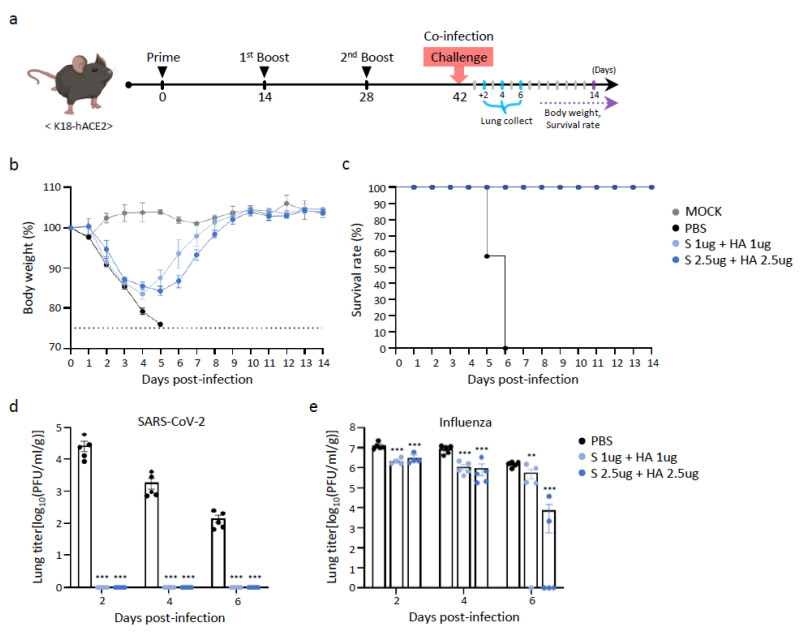

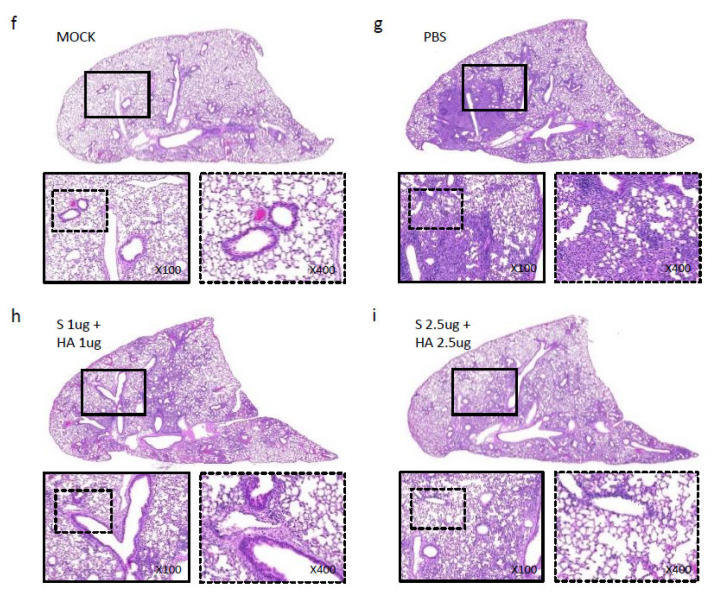
Protective efficacy of a multivalent BLP vaccine candidate against co-infection in K18 hACE2 mice. (**a**) Experimental design showing vaccine immunization and co-infection. Mice were intramuscularly administered a mixture of iLact-tSP and iLact-tHA (1 or 2.5 µg) three times at two-week intervals. Two weeks after the 2nd booster immunization, mice were co-infected with influenza (1 × 10^5^ PFU) and SARS-CoV-2 (1 × 10^5^ PFU). (**b**) Body weight changes and (**c**) survival rates were monitored for 2 weeks (*n* = 7) post-co-infection. Weight loss exceeding 25% of the initial body weight was deemed an experimental endpoint. Mice (*n* = 5) were euthanized on 2, 4, and 6 DPI, and the viral titers of (**d**) influenza and (**e**) SARS-CoV-2 in the lungs were determined using a plaque assay in MDCK cells. Hematoxylin and eosin (H&E) staining sections showed histopathological changes in the mouse lungs 4 days after co-infection. (**f**) Non-infected mice were sacrificed and the lungs were excised as normal controls. (**g**) Mice treated three times with PBS and (**h**) mice immunized three times with HA 1 µg + S 1 µg or (**i**) HA 2.5 µg + S 2.5 µg were co-infected. Five mice per group were sacrificed 4 days after co-infection for H&E staining. Data are expressed as the mean ± SEM. The statistical significance of differences between groups was calculated by two-way ANOVA with Tukey’s multiple comparisons test (*p* < 0.05, ** *p* < 0.01, *** *p* < 0.001). Dotted lines reflect the assay limit of quantitation.

We evaluated whether BLP combined with SARS-CoV-2 S and influenza HA proteins could serve as an effective vaccine candidate, even in cases of co-infection with SARS-CoV-2 and influenza viruses. Following three rounds of BLP immunization, body weight changes and survival were observed for up to 14 DPI following influenza (A/Victoria/2570/2019) and SARS-CoV-2 (Omicron BA.1) infections. In the PBS-treated group challenged with both SARS-CoV-2 and influenza virus, mice began to die at 5 DPI, with all mice succumbing by 6 DPI. In contrast, the BLP-vaccinated groups exhibited weight loss until 4 DPI but subsequently recovered, reaching weight levels comparable to those of PBS-treated mice by 9 DPI (Figure 6b).

After co-infection with both SARS-CoV-2 and influenza, viral titers in the lung tissue were measured, revealing results similar to those observed when each virus was individually challenged earlier (Figure 6d,e). In the PBS-treated control group, influenza virus titers remained high until 6 DPI. However, in the BLP-immunized group, the viral titer decreased significantly. Similarly, SARS-CoV-2 viral titers persisted for up to 6 DPI in the PBS-treated group, whereas they were undetectable at all time points following co-infection in the BLP-immunized group. Histopathological examination using H&E staining showed that the lungs of the BLP vaccine-immunized mice (Figure 6h,i) exhibited fewer pathological changes than those of PBS-treated mice (Figure 6g). The lungs of the mice immunized with HA 2.5 µg and S 2.5 µg BLP vaccines appeared to show intact alveoli, almost similar to that of the normal mice (Figure 6f). These results demonstrate that BLP-expressing SARS-CoV-2 S and influenza HA proteins provide superior protection not only against single-virus infection but also in cases of co-infection with both SARS-CoV-2 and influenza.

## 4. Discussion

This study highlights the significant potential of plant-based platforms, particularly *Nicotiana benthamiana*, for producing recombinant proteins used in vaccine development. The use of *N. benthamiana* offers several distinct advantages, making it a particularly attractive system for large-scale vaccine production. Firstly, its rapid growth cycle allows for quick biomass production, which is crucial during emergencies like pandemics where time is of the essence [30]. In addition, the ease of genetic manipulation in *N. benthamiana* enables the production of complex proteins that are difficult to express in other systems. This versatility makes plant-based systems viable and scalable, as the infrastructure required for growing plants is simpler than that needed for mammalian cell cultures or other bioreactor-based systems. Moreover, the use of plants as bioreactors inherently reduces the risk of contamination by human or animal pathogens, enhancing the safety profile of the resulting vaccines [31]. This characteristic is particularly important, as contamination in traditional vaccine production systems can lead to serious public health issues.

In the context of vaccine design, this study employed a strategy of coating bacterium-like particles (BLPs) derived from *Lactobacillus sakei* with S and HA proteins. This method mimics the structural features of bacteria during infection, enhancing vaccine immunogenicity [32]. The idea of using cBLPs aligns with the growing body of research exploring bacteria or BLPs as carriers or adjuvants in vaccine development [33]. These particles can enhance the immune response by presenting antigens in a manner that mimics natural infections, triggering a more robust and effective immune response. The findings of this study underscore the effectiveness of BLPs in eliciting strong immune responses, even in the absence of additional adjuvants. This is a significant observation because it positions BLPs as promising candidates for vaccine development, potentially reducing the need for traditional adjuvants that can cause adverse reactions [34]. This approach could lead to the development of vaccines that are not only more effective but also safer and more tolerable for a broader range of populations, including those sensitive to adjuvants.

Specifically, for SARS-CoV-2, the S protein was selected as the primary antigen based on its superior immunogenicity compared to other subunits, such as the receptor-binding domain (RBD) or S1 protein [35]. The full-length S protein offers distinct advantages, particularly because it contains conserved T-cell epitopes within its S2 domain, which have the potential to provide cross-protection against emerging viral variants—an essential feature considering the ongoing viral evolution [36]. Continuous mutations in the virus present a significant challenge to vaccine efficacy, as new variants may partially evade the immune responses generated by earlier vaccines. Therefore, the inclusion of a full-length S protein—which may elicit broader immune responses—is a strategic choice that could enhance the long-term effectiveness of the vaccine [37]. This approach could potentially reduce the need for frequent updates to vaccine composition—a challenge currently faced in the fight against COVID-19.

Similarly, the HA protein from the A/California/07/2009 H1N1 influenza strain was chosen for its proven ability to induce neutralizing antibodies (nAbs) that can protect against influenza infections [38,39,40]. The HA protein is a well-established target in influenza vaccine development, and its inclusion in this study is supported by extensive research. HA-based vaccines can trigger effective immune responses and confer protection against various strains of influenza viruses [41]. The selection of the HA protein from the 2009 H1N1 pandemic strain is particularly relevant, given the need for protecting against a broad spectrum of influenza viruses, including those with pandemic potential. This choice ensures that the vaccine offers protection against seasonal influenza, as well as novel strains that may emerge in the future.

This study investigated a multivalent vaccine approach combining both the SARS-CoV-2 S protein and influenza HA protein to assess its potential for generating broad-range protective immunity. Experimental data demonstrated that the multivalent BLP vaccine elicited strong humoral and cellular immune responses in both BALB/c and K18-hACE2 mouse models [42]. Notably, the vaccine-induced high titers of neutralizing antibodies against both SARS-CoV-2 and influenza offer effective protection against co-infection. This is a critical finding, as co-infection with respiratory viruses like influenza and SARS-CoV-2 can exacerbate disease severity and complicate treatment [43]. By protecting against both pathogens, multivalent vaccines can significantly reduce the burden of respiratory infections, particularly in vulnerable populations.

Furthermore, this study emphasized the practical implications of using plant-based expression systems to produce vaccine antigens. The scalability of protein extraction from plant leaves, coupled with the effectiveness of the vaccine produced using this method, highlights its potential for cost-effective and rapid vaccine production. This is particularly critical during pandemics when the ability to quickly scale up vaccine production can save countless lives [44]. Moreover, the use of plants as bioreactors offers environmental benefits, as they require fewer resources and generate less waste than traditional vaccine production methods. This aspect of sustainability will likely become increasingly important as the world faces the dual challenges of global health and environmental conservation.

## 5. Conclusions

The BLP-based multivalent vaccine prepared in this study is a promising strategy not only for combating SARS-CoV-2 and influenza individually but also for addressing the challenge of co-infection. The vaccine’s ability to induce robust immune responses and confer protection in animal models supports its further development for potential human use. Additionally, the innovative use of plant-based systems for vaccine production could revolutionize the field by offering scalable, safe, and environmentally sustainable alternatives to traditional methods. As research progresses, this approach could lead to the development of vaccines that are not only highly effective but also accessible to the global population, particularly in low-resource settings where traditional vaccine production is less feasible.

## Data Availability

The data presented in this research are available on request from the corresponding author.

## Supplementary Materials

The following supporting information can be downloaded at: https://www.mdpi.com/article/10.3390/vaccines13030254/s1, Figure S1: Estimation of S and HA proteins bound to cBLP.
