# Supplementary material for: Development of Plant-Based Multivalent Vaccine Candidates for SARS-CoV-2 and Influenza Virus Using Inactivated Lactococcus
Source: Vaccines (Basel). 2025 Feb 27;13(3):254. doi: 10.3390/vaccines13030254 (PMC11945824; doi:10.3390/vaccines13030254)
Supplement: Supplementary file 1 [file vaccines-13-00254-s001.zip › vaccines-3415454-supplementary.pdf]

Development of Plant-based Multivalent Vaccine Candidates for SARS-CoV-2 and Influenza Virus Using Inactivated *Lactococcus*

Supplementary Material

Dong-Sook Lee, Hasanul Banna, HeeYeon Kim, Md Rezaul Islam Khan, Hai-Ping Diao, Shi-Jian Song, Young-Eui Kim, Haeji Kang, Jungsang Ryou, Joo-Yeon Lee, Jang-Hoon Choi, Inhwan Hwang* and Sehee Park*

Supplementary Figure

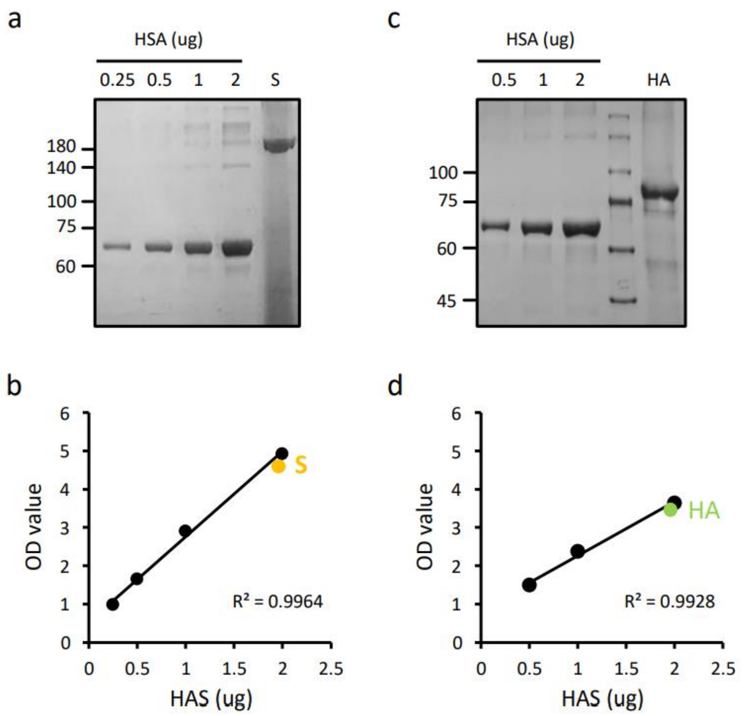

Figure S1. Estimation of S and HA proteins bound to BLP.

The suspension of (a) BLP-S or (b) BLP-HA complex (10 ml each), together with the indicated amount of HSA as a reference, was separated by SDS-PAGE, and the gels were stained with Coomassie brilliant blue. The intensity of the protein bands was measured using LAS3000 software. A standard curve of HSA was prepared for both (c) S protein and (d) HA samples. The amounts of S and HA were estimated using the HSA standard curve.

Abbreviations: S, SARS-CoV-2 Omicron BA.1 variant spike protein; HA, H1N1 virus hemagglutinin; BLP, bacterium-like particle; BLP, bacterium-like particle; HAS, human serum albumin; SDS-PAGE, sodium dodecyl sulfate-polyacrylamide gel electrophoresis.
